# Non-Motor Symptoms in *PLA2G6-*Associated Dystonia-Parkinsonism: A Case Report and Literature Review

**DOI:** 10.3390/jcm11061590

**Published:** 2022-03-13

**Authors:** Lydia Vela-Desojo, Daniele Urso, Mireia Osuna-López, Janet Hoenicka

**Affiliations:** 1 Hospital Fundación Alcorcón, 28922 Madrid, Spain; 2 HM Centro Integral en Neurociencias (CINAC), University Hospital HM Puerta del Sur, 28938 Madrid, Spain; daniele.urso@kcl.ac.uk; 3 Department of Neuroscience, Institute of Psychiatry, Psychology and Neuroscience, De Crespigny Park, King’s College London, London SE5 8AF, UK; 4 Laboratory of Neurogenetics and Molecular Medicine, Institut de Recerca Sant Joan de Déu, C/Santa Rosa 39-57, Esplugues de Llobregat, 08950 Barcelona, Spain; mireia.osuna@gmail.com; 5 Centro de Investigación Biomédica en Red de Enfermedades Raras (CIBERER), 08950 Barcelona, Spain

**Keywords:** PLAN-DP, *PLA2G6*, NMS, premotor, dementia, psychiatric symptoms

## Abstract

*PLA2G6*-dystonia-parkinsonism (PLAN-DP) is characterized by levodopa responsive parkinsonism and dystonia. While neuropsychiatric symptoms and early cognitive decline are also common in this entity there is little information regarding other non-motor symptoms (NMS). Here, we describe a 26-year-old patient with PLAN-DP whose motor symptoms were preceded by mild cognitive impairment and anxiety, and who developed many other NMS as the disease evolved. Furthermore, we reviewed the NMS described in all the PLAN-DP patients published to date. A total of 50 patients with PLAN-DP were identified, 42 of whom developed NMS and in 23 of these cases, NMS preceded the motor symptoms of the disease. Neuropsychiatric symptoms dominated the premotor phase of this condition and cognitive impairment/dementia was the most prevalent NMS. Other NMS were reported infrequently like sleep disorders, autonomic symptoms, pain and hyposmia, and mostly as the disease evolved. NMS are very frequent in PLAN-DP and they may appear before diagnosis or during the course of the disease. Neuropsychiatric symptoms and cognitive decline are the most frequent NMS. The appearance of neuropsychiatric symptoms like depression, anxiety or personality changes prior to a diagnosis of parkinsonism in younger individuals might suggest the presence of *PLA2G6* gene mutations.

## 1. Introduction

Non-motor symptoms (NMS) are highly prevalent in idiopathic [1] and familial Parkinson′s disease (PD) [2,3]. *PLA2G6-*dystonia-parkinsonism (PLAN-DP) is characterized by adult-onset levodopa-responsive parkinsonism with dystonia, and it is associated with several mutations in the *PLA2G6* gene [4]. *PLA2G6* encodes the calcium-independent phospholipase A_2_β (iPLA_2_β), which hydrolyzes membrane phospholipids, thereby regulating membrane homeostasis and generating potent lipid second messengers involved in cell proliferation, mitochondrial dynamics, Ca^2+^ signaling and apoptosis [5]. Biallelic *PLA2G6* mutations have been also associated with infantile and atypical neuroaxonal dystrophies [4]. The NMS in PLAN-DP have not been well defined, except for the presence of neuropsychiatric symptoms like depression, psychosis and early cognitive decline or dementia [6,7]. A recent review synthesized the clinical and genotypic data available in the literature on parkinsonism with onset in individuals younger than 21 years of age [8], and it included 16 patients carrying a *PLA2G6* mutation. Here, we report the case of a young patient with parkinsonism who was homozygous for the p.H479D (c.1435C˃G) mutation in the *PLA2G6* gene. The motor symptoms in this patient were preceded by severe anxiety and he developed throughout the disease several NMS. In addition, we have reviewed the clinical data on all the patients with PLAN-DP published to date, describing all their NMS.

## 2. Case Presentation

The patient, a 26-year-old male, was delivered after a normal pregnancy. Psychomotor milestones were achieved at a normal age, although since childhood he exhibited intellectual disability and underperformed at school. At 24 years of age, he was diagnosed with anxiety and social phobia, and he was prescribed alprazolam after having an anxiety attack. At age 26, his psychiatrist considered that evaluation by a neurologist was warranted. Although neither the patient nor his family had noticed any neurological problem, physical examination revealed hypomimia, a hypophonic voice, asymmetric bradykinesia, rigidity and a mildly dystonic posture in his left extremities. A DaTscan indicated normal activity in the nigrostriatal pathway, but the patient was prescribed levodopa/carbidopa. Three months later, the patient´s movements had improved strikingly, his gait was normal, he did not have rigidity or dystonia, and only mild bradykinesia was present in the left extremities. However, a year after starting on levodopa, he developed severe motor fluctuations and dyskinesias. A new DaTscan at that time revealed a severe loss of dopamine transporters, which was particularly prominent in the right putamen. As a result, the patient’s medication was modified to control his symptoms. A neuropsychological evaluation was also performed and it revealed cognitive impairment not interfering with daily life activities. In the following years, the motor and non-motor fluctuations (severe visceral pain), and the dyskinesias became difficult to control. The patient also developed constipation, urinary incontinence, visual hallucinations, sleep problems with suggestive rapid eye movement (REM) behavior disorder, anxiety, gait freezing and severe instability associated with frequent falls. Indeed, seven years after the first visit, he was wheelchair-bound and demented, with dysphagia and emotional lability. Magnetic resonance imaging (MRI) showed severe diffuse cerebral atrophy with iron deposits in the globi pallidi. We have followed up the patient for 14 years and, at the time of writing, he is still alive.

PARK *loci* genes were analyzed in a genetic panel at 50x coverage. After a bioinformatics analysis, filtering and confirmation by the Sanger method, we identified the non-synonymous single nucleotide variation (SNV) in the *PLA2G6* gene c.1435C>G (p.His479Asp; rs1235695530, gnomAD browser frequency: 0), as well as the homozygous synonymous SNV c.1494C>T (p.Ile498Ile; rs983066224, gnomAD browser frequency: 0). The in silico study of p.His479Asp using the Combined Annotation Dependent Depletion (CADD) [9] showed a high C-score (21.2), thus suggesting a high likelihood of a deleterious effect. Table 1 shows other pathogenicity predictors used to evaluate p.His479Asp. In addition, we analyzed the *PLA2G6* promoter regions, spanning 2900 base pairs (bp) of the 5′ UTR (untranslated region), and we also analyzed 700 bp of the 3′ UTR of this gene. This analysis failed to identify any further variations. Significantly, the patient′s parents were heterozygous for the two SNVs, confirming Mendelian segregation (Supplementary Figure S1).

## 3. Literature Search and Selection

A search of publications between January 2009 and November 2019 was carried out in the PubMed and EMBASE international databases using the following terms: dystonia OR Parkinson OR dystonia-parkinsonism OR parkinsonism AND *PLA2G6* OR *NBIA2B* OR PLAN OR *PARK-14*. We also searched the national Dialnet and IBECS databases using the same terms in Spanish. We found 67 articles, 25 of which described one or more patients [4,6,7,15,16,17,18,19,20,21,22,23,24,25,26,27,28,29,30,31,32,33,34,35,36,37]. We also included another article published after November 2019 [38]. The criteria for inclusion were: 1, patients with levodopa-responsive parkinsonism in whom other symptoms like ataxia, myoclonus or spasticity were not the predominant symptoms during the evolution of the disease; 2, patients with homozygous or compound heterozygous *PLA2G6* gene mutations; 3, patients in which sufficient clinical information was available. If patients were published in more than one article, only the one with the most complete description was chosen.

We carefully read all of the articles to extract the clinical information for the patients and we included all the NMS described in the analysis: cognitive decline or dementia, depression, psychotic symptoms, autonomic symptoms, anxiety, sleep disorder and olfactory impairment. We chose the term “psychotic symptoms” to reflect all types of hallucinations, paranoia and delusion. Sleep problems included restless legs syndrome, REM sleep behavior disorder or daytime sleepiness. Autonomic symptoms comprised any of the following: urinary frequency, urgency or incontinence; sweating problems; constipation; and orthostatic hypotension. NMS may precede the movement disorder, they may occur concomitantly or they may appear during the progression of the disease. We considered them as premotor if they preceded or if they were present at the time of PD diagnosis. For statistical analysis, the quantitative results are shown as the mean and standard deviation (SD), or as the median and interquartile range (IQR) if the data did not follow a normal distribution. The frequencies were expressed as the absolute values “n” and as percentages.

## 4. Results of the Literature Review

We included 50 patients in the analysis, 49 from the literature and the patient described here. Of these 50 patients, 28 (56%) were men and they had a mean age of 26 years old at PD diagnosis (IQR 22-31). Of these, 80% were Asian (India/Pakistan 5, 10%; Far East 10, 20%; or Middle East 25, 50%) and 20% were Caucasian (10). Almost all the patients had at least one NMS (84%) and 23 patients (46%) had a premotor NMS (median 2, range 1–3), the most common of which were depression (24%), psychotic symptoms (16%) and cognitive impairment (14%). Personality or behavioral changes (10%) and anxiety (10%) were also frequent, and thus, neuropsychiatric symptoms dominated the premotor phase of this condition. NMS were first described 3.5 (0–20) years before PD diagnosis in the 15 patients in whom this data was available. There were also 40 patients (80%) in whom at least one NMS developed after the diagnosis of parkinsonism (median 2.5, range 2–3). Cognitive decline or dementia was the most frequent symptom, evident in 62% of patients, while psychosis (34%) and autonomic symptoms (36%) were also very common (Table 2). Sleep problems were described in 1 patient before diagnosis and in 4 patients during the evolution of the disease. Hyposmia was only described in one patient, while 3 patients had NM fluctuations (pain, dysphagia, neuropsychiatric symptoms, etc.). Dementia was most frequent in those patients who were younger than 30 years at diagnosis (79.3% vs 50%).

Collectively, the patients showed significant variability in their premotor NMS and in the main clinical symptoms, including motor complications and NMS, with a limited phenotype/genotype correlation. However, we found in patients with more NMS a higher prevalence of c.4C>A (p.Q2K) (six patients) and c.2222G>A (p.R741Q) (four patients), which are located at the amino- and carboxy-ends of *PLA2G6* protein, respectively.

## 5. Discussion and Conclusions

We describe here the clinical features, mainly the NMS, of a new patient with adult-onset PLAN-DP, and we have reviewed the NMS of all the patients described in the literature from 2009 to date. The patient described here was diagnosed with parkinsonism at the age of 26, although he had mild cognitive impairment since childhood. Indeed, in the previous two years, he had been treated by a psychiatrist due to high levels of anxiety and social phobia. Furthermore, throughout the disease, he developed other NMS, such as urinary incontinence, visual hallucinations, sleep problems, emotional lability and dementia, as well as symptoms like fluctuating visceral pain, not previously described in these patients.

Taking into account the information on all the PLAN-DP patients published to date, we found that NMS are very frequent in this condition, evident in more than 80% of patients. NMS preceded the diagnosis of parkinsonism in 46% of patients [4,16,18,23,24,25,26,27,28,29,30,31,32,33,38], sometimes even 20 years earlier. Unlike idiopathic Parkinson´s disease (iPD) in which hyposmia, REM sleep behavior disorder, depression and constipation are the most frequent premotor symptoms [39], neuropsychiatric symptoms dominate the clinical landscape in *pre-motor* PLAN-DP. The functional impact of premotor symptoms was not assessed in PLAN patients, yet considering the severity and nature of these symptoms, their functional impact would probably have been stronger than in iPD [38]. There is not much information about premotor symptoms in other genetic parkinsonisms, except in patients with *LRRK2* mutations in whom the loss of smell, depression, constipation, and excessive daytime sleepiness were reported at the time of diagnosis in more than 40% of patients [40].

In this review, impaired cognition was the most frequent NMS, evident in 64% of patients [4,7,16,20,21,22,23,24,25,26,28,29,32,33,37,38]. This phenomenon was detected before PD diagnosis usually manifested as school failure [4,18,24,25,29] and after the diagnosis of parkinsonism. Memory complaints are frequent in early iPD [39], although cognitive changes are usually subtle [41]. Cognitive impairment is more frequent with the progression of the disease, affecting up to 40% of patients [42]. Cognitive deficits are not usually associated with genetic parkinsonisms like *Parkin* and *DJ-1* [3], even though it may develop in 13.5–23% of leucine-rich repeat kinase 2 (*LRRK2)*-linked PD patients and it is very frequent in parkinsonism associated with mutations in PTEN-induced putative kinase 1 (*PINK-1*) [43] in and in *SNCA* mutations and multiplications, mainly in those who are carriers of *SNCA* triplications, as well as in parkinsonism associated with mutations in *GBA* [3].

A recent review indicated that cognitive impairment was evident in 92% of patients under 21 years of age with mutations in *PLA2G6* [8]. We found that dementia was more common in patients younger than 30 years of age. Because there are other phenotypes associated with *PLA2G6*, such as infantile neuroaxonal dystrophy (INAD) in which symptoms and dementia are very frequent [4], our data might support the idea that there is a continuum in the clinical spectrum of this entity, as indicated previously [44].

Psychotic symptoms were also very frequent in PLAN-DP patients, with 16% of patients having them as a premotor manifestation and in 34% developing such symptoms during the course of the disease. Psychotic symptoms appear in up to 40% of patients with iPD [45], although they are less frequent in other genetic parkinsonisms except for *PINK1*-linked disease and parkinsonism associated with *GBA* mutations [2,43].

Autonomic symptoms were present in 34% of PLAN patients, although they were probably under-reported due to the presence of more severe symptoms. The prevalence of dysautonomia in iPD is higher than 50% and it appears to be associated with 15% of *LRRK2* and 40% of *SNCA* mutations in genetic parkinsonisms [3].

Mutations in more than 70 different genes have been associated with early-onset parkinsonism or may feature parkinsonism as part of their phenotypic spectrum. The combination of clinical findings with the abnormal imaging pattern and presence or absence of systemic signs can refine the diagnostic suspect and lead to the identification of these disorders [8]. In PLAN-DP, cerebellar atrophy and pallidal iron deposition, as observed in this case, have been reported. However, these imaging signs can be found in other disorders. Intellectual disability of different degrees of severity that preceded the onset of parkinsonism can be found also in patients with *ATP6AP2, PGK1, PTRHD1* and *RAB39B* mutations, most of which develop parkinsonism after a period of non-progressive intellectual disability. Neuropsychiatric symptoms are also reported in *ATP13A2* and *FBXO7* mutations [8]. Notably, patients with 22q11.2 deletion syndrome may present variable intellectual disability, psychosis and neuropsychiatric manifestations with later development of levodopa-responsive parkinsonism. This diagnosis has management implications because of the associated systemic manifestations of DiGeorge syndrome such as hypocalcaemia, autoimmune thyroiditis and structural cardiac abnormalities [46].

This is the first time the status of all NMS in patients with PLAN-DP has been reviewed. Recently, a review summarized the relationship between phenotype and genotype in *PLA2G6*-Related Parkinsonism [47]. However, the review did not focus on NMS. We have tried to show the prevalence of these NMS and characterize them. However, it is important to consider some of the limitations of the study. The information on NMS associated with *PLAN-DP* is based only on case reports. While the results are based on the clinical description of every patient in each publication, usually only significant information is given and it is likely some data is missing. The presence of more severe and disabling NMS, such as cognitive impairment or psychosis, probably masks other “minor” symptoms like constipation or hyposmia. Furthermore, it is possible that patients or their families were unaware of some of the NMS or they may even not consider them important, such that they are not often declared at hospital consultations because they did not consider them to be related to their disease, as occurred with iPD [42]. Thus, it would be important to systematically assess patients with PLAN-DP about NMS, for example using the NMS questionnaire [42] in order to identify these symptoms and to treat them where possible.

NMS are very frequent in PLAN-DP and they may appear before the diagnosis or during the course of the disease. Neuropsychiatric symptoms and cognitive decline are the most frequent NMS, evident before diagnosis and during the course of the disease. The presence of neuropsychiatric symptoms like depression, anxiety or personality changes prior to the motor onset of parkinsonism in younger people might suggest the presence of *PLA2G6* gene mutations.

## Figures and Tables

**Table 1:** Pathogenicity Predictors for the *PLA2G6* variant p.His479Asp.

ClinVar	ACMG ^a^ (23 February 2022)		DANN ^b^	SIFT ^c^	PROVEAN ^d^	Mutation Assessor ^e^	Mutation Taster ^f^
	**Verdict**	**Rules**					
Likely pathogenic (7 August 2020)	Pathogenic	PM2 Strong PP2 Supporting PP5 Strong	0.9906	Damaging 0.006	Neutral −1.413	Medium 1.995	Polymorphism 0.7776

Reference sequence: NM_003560.4. ^a^ ACMG. American College of Medical Genetics [10,11]. ^b^ DANN. The value range is 0 to 1, with 1 given to the variants predicted to be the most damaging. ^c^ SIFT scores range between 0 (pathogenic) and 1 (benign). ^d^ PROVEAN. If the score is equal to or below a predefined threshold (−2.5), the protein variant is predicted to have a “deleterious” effect. If the score is above the threshold, the variant is predicted to have a “neutral” effect. [12]. ^e^ Mutation Assessor. Range −5.135 to +6.49. [13]. ^f^ MutationTaster [14].

**Table 2:** NMS in patients with *PLA2G6*-associated dystonia-parkinsonism.

	Premotor NMS	NMS during PD Evolution
Patients, N (%)	23 (46%)	42 (84%)
Depression	12 (24%)	10 (20%)
Anxiety	5 (10%)	5 (10%)
Personality or behavioral changes	5 (10%)	6 (12%)
Psychosis	8 (16%)	17 (34%)
Impulsivity		4 (8%)
Cognitive impairment/Dementia	7 (14%)	31 (62%)
Emotional lability		7 (14%)
Autonomic symptoms	4 (8%)	18 (36%)
Sleep problems	1 (2%)	4 (8.5%)
Dysphagia		7 (14%)
Hyposmia	1 (2%)	
Pain		1 (2%)
NM fluctuations		3 (6%)

## Data Availability

The data that support the findings of this study are available from the corresponding author, upon reasonable request.

## Supplementary Materials

The following are available online at https://www.mdpi.com/article/10.3390/jcm11061590/s1, Figure S1: Sanger Sequencing results of the *PLA2G6* variant p.His479Asp (c.1435C>G). A. Patient; B and C. Progenitors.
