# Supplementary material for: Non-Motor Symptoms in PLA2G6-Associated Dystonia-Parkinsonism: A Case Report and Literature Review
Source: J Clin Med. 2022 Mar 13;11(6):1590. doi: 10.3390/jcm11061590 (PMC8950520; doi:10.3390/jcm11061590)
Supplement: Supplementary file 1 [file jcm-11-01590-s001.zip › jcm-1612007-supplementary.pdf]

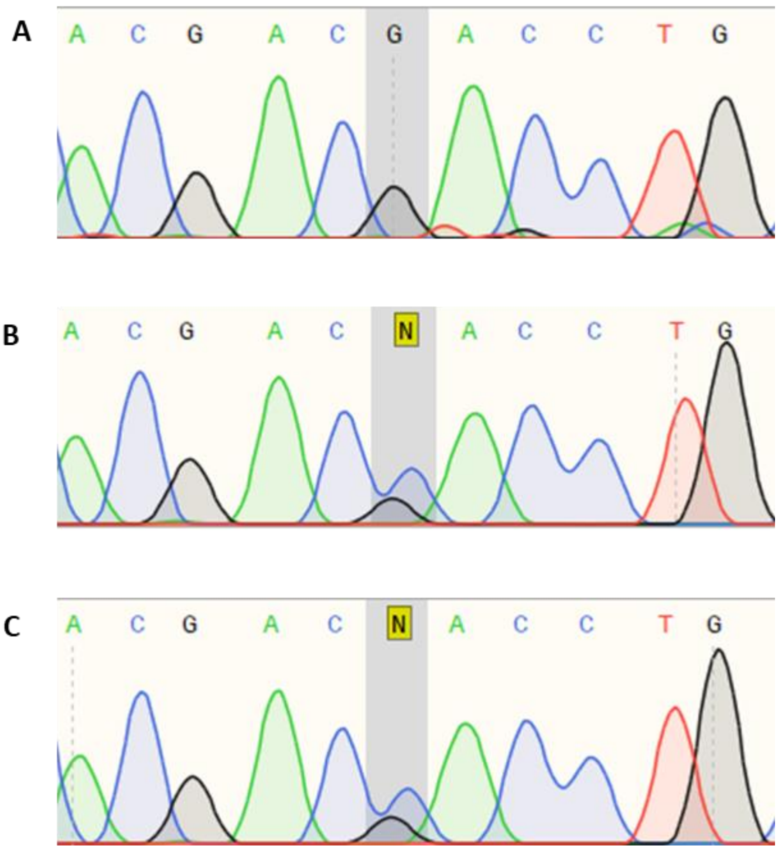

Figure S1. Sanger Sequencing results of the *PLA2G6* variant p.His479Asp (c.1435C>G). A. Patient; B and C. Progenitors.
